# Basal and Dynamic Leptin Secretion: Association with Cardiometabolic Risk and Body Weight Trajectories in African-Americans and European-Americans

**DOI:** 10.3389/fendo.2018.00012

**Published:** 2018-01-26

**Authors:** Sotonte Ebenibo, Chimaroke Edeoga, Ibiye Owei, Sam Dagogo-Jack

**Affiliations:** ^1^Division of Endocrinology, Diabetes and Metabolism, University of Tennessee Health Science Center, Memphis, TN, United States

**Keywords:** leptin regulation, glucocorticoids, race/ethnicity, weight trajectories, cardiometabolic risk

## Abstract

**Background:**

Fasting plasma leptin levels reflect fat mass, but dynamic leptin responses to secretagogues, and the influence of race/ethnicity, have not been well studied. Here, we compared basal and stimulated leptin levels in relation to cardiometabolic risk and weight trajectories in black and white subjects.

**Subjects and methods:**

We studied 254 (127 black and 127 white) normoglycemic adults enrolled in the Pathobiology of Prediabetes in a Biracial Cohort (POP-ABC) study. At baseline and annually, POP-ABC participants underwent physical examination, oral glucose tolerance test, and measurements of body fat (dual energy X-ray absorptiometry), fasting plasma leptin, insulin, cortisol, lipids, and leptin secretory response to single-dose (2 mg) dexamethasone (dex). The interactions among basal and stimulated leptin and changes in adiposity/cardiometabolic measures during the ensuing year were then analyzed.

**Results:**

The mean (±SD) fasting leptin level (50.6 ± 47.7 vs. 39.5 ± 37.6 ng/mL, *P* = 0.004) and body mass index (BMI) (31.9 ± 7.14 vs. 29.0 ± 7.66 kg/m^2^, *P* = 0.0043) were higher in black women vs. white women, but similar in black men vs. white men (leptin: 12.4 ± 2.07 vs. 11.1 ± 1.40 ng/mL; BMI: 29.4 ± 7.68 vs. 28.1 ± 4.23 kg/m^2^). The peak leptin response to dex (~200% baseline) did not differ significantly by gender or race. Total body fat correlated positively with fasting leptin (*r* = 0.81, *P* < 0.0001) and inversely stimulated leptin levels (*r* = −0.26, *P* < 0.0001). Fasting leptin was unrelated to 1-year change in weight or fat mass, whereas stimulated leptin levels were significantly associated with 1-year trajectories in weight (*P* = 0.0016) and total fat mass (*P* = 0.0035). Stimulated leptin levels also had significant interactions with insulin sensitivity (homeostasis model of insulin resistance, *P* = 0.01), triglycerides (*P* = 0.0078), fasting glucose (*P* = 0.027), systolic blood pressure (*P* = 0.037), and high-sensitivity C-reactive protein (*P* = 0.027).

**Conclusion:**

We found no significant ethnic disparities in basal or dynamic leptin secretion in relation to adiposity. Fasting leptin levels were not associated with 1-year weight change, while stimulated levels showed weak though significant association with 1-year weight change.

## Introduction

Leptin regulates body weight and influences metabolic processes in rodents (1–3) and human beings (4–8). The analysis of plasma leptin levels in relation to human metabolic pathophysiology has focused largely on specimens collected under basal (fasting) conditions (9–13). The findings from such studies indicate that fasting leptin levels are directly correlated with measures of adiposity (9–11) in lean and obese subjects as well as in persons with diabetes (14). We and others have reported that administration of glucocorticoids results in significant increases in plasma leptin levels (15–19). Others have reported that prolonged insulin infusion also increases plasma leptin levels (20). Thus, glucocorticoids and insulin can be deployed as leptin secretagogues to probe the metabolic physiology and pathophysiology of dynamic leptin secretion. Given the many drawbacks of insulin (the need for injection, risk of hypoglycemia, and obligate need to coadminister glucose), oral glucocorticoid is a more convenient secretagogue for studying dynamic leptin secretion. The previous reports had utilized different doses of methylprednisolone (15), dexamethasone (dex) (16, 17), or hydrocortisone (18, 19) to demonstrate glucocorticoid-induced leptin secretion. In this report, we investigated leptin responses to a single, 2-mg dose of dex in African-American (black) and European-American (white) men and women with a broad range of adiposity measures. Our data indicate that augmentation of plasma leptin levels in response to a single oral dose (2 mg) of dex is a highly conserved trait that may convey information regarding cardiometabolic risk status and weight trajectories.

## Materials and Methods

### Study Subjects

We studied 254 (127 black, 127 white) non-diabetic participants enrolled in the Pathobiology of Prediabetes in a Biracial Cohort (POP-ABC) study (21, 22). The POP-ABC participants had one or both biological parents with type 2 diabetes (T2D) and met the following additional inclusion criteria: age 18–65 years; non-Hispanic white or non-Hispanic black race/ethnicity status; no evidence of diabetes; normal fasting plasma glucose (FPG) [<100 mg/dL (5.6 mmol/L)] and normal glucose tolerance [2-hr plasma glucose (2hrPG) < 140 mg/dL (7.8 mmol/L) during a 75-g oral glucose tolerance test (OGTT)]; and good overall health, as previously described (21, 22). Persons using antidiabetes medications or other medications known to alter glucose metabolism or body weight were ineligible for enrollment. Also excluded were persons enrolled in active weight loss programs, using behavioral, pharmacological, surgical, or combined modalities (21, 22).

At baseline and annually, POP-ABC participants underwent physical examination, OGTT, and measurements of body fat (dual energy X-ray absorptiometry), blood lipids and other analytes, and leptin secretion. The POP-ABC study protocol was approved by the University of Tennessee Health Science Center Institution Review Board. All subjects gave written informed consent before the initiation of the study, which was conducted in accordance with the principles of the Declaration of Helsinki.

### Leptin Secretion Test

Basal and stimulated leptin secretion was assessed as previously described (14), with dex dose reduction from 4 to 2 mg. In brief, on day 1 of the test, participants arrived at the General Clinical Research Center (GCRC) in the fasting state for baseline blood sampling at 0800 h and were given a single 2-mg tablet of dex. Between 11:00 p.m. and midnight on day 1, participants ingested the 2-mg dose of dex and returned to the GCRC on day 2 for fasting blood sampling at 8:00 a.m. (8-h sample). After that, participants had their usual breakfast and lunch and returned for final blood sampling at 4:00 p.m. on day 2 (16-h sample). Study subjects were given written instructions to maintain their usual dietary and physical activity habits and avoid strenuous exercise for at least 3 days before the test.

### Biochemical Measurements

Plasma glucose was measured with a glucose oxidase method (Yellow Spring Instruments Co., Inc., Yellow Spring, OH, USA). Plasma insulin, cortisol, and high-sensitivity C-reactive protein (hsCRP) levels were measured immunochemically using commercial kits. Plasma leptin was measured by ELISA kit (Millipore, St. Charles, MO, USA). The leptin assay captured total circulating human leptin, with a sensitivity of 0.5 ng/mL and within-batch and between-batch coefficients of variation of <5%, respectively. Fasting plasma lipid profiles were measured in a contract clinical laboratory. The homeostasis model of insulin resistance (HOMA-IR) and homeostasis model of beta-cell function (HOMA-B) were derived from fasting glucose and insulin values (23).

### Statistical Analysis

Data are reported as means ± SD unless SEM is specified. Significance level was set as *P* < 0.05. Differences between groups were analyzed using unpaired *t*-tests and analysis of variance (ANOVA) for continuous variables and chi-squared test for discrete variables. General linear regression models were used to analyze the relationship between basal and stimulated plasma leptin levels and adiposity and cardiometabolic measures. A percentile plot was generated to display the spectrum of stimulated peak leptin responses for the entire study cohort, and to enable sensitivity analyses. All statistical analyses were conducted using SAS statistical software, version 9.3 (SAS Institute Inc., Cary, NC, USA). The level of significance was set at *P* < 0.05.

## Results

### Cohort Characteristics

Table 1 shows the baseline characteristics of black and white participants. The gender distribution was similar in both groups. The mean age was lower and body mass index (BMI) higher in black than white subjects, but total and trunk fat mass were similar in the two groups. The ethnic difference in BMI was driven by higher values in black women: 31.9 ± 7.14 vs. 29.0 ± 7.66 kg/m^2^, *P* = 0.0043; the BMI was not significantly different in black men vs. white men (29.4 ± 7.68 vs. 28.1 ± 4.23 kg/m^2^) FPG, and serum triglyceride levels were higher in white than black participants, although glucose and lipid values were well within the normal ranges in entire cohort.

**Table 1:** Baseline characteristics of study participants by ethnicity.

	Black subjects	White subjects	*P*-value
Number	127	127	
Female/male	88/39	86/41	0.78
Age (years)	42.5 ± 10.3	46.6 ± 10.5	0.0002
Body mass index (kg/m^2^)	31.2 ± 7.37	28.8 ± 6.81	0.001
Fasting plasma glucose (mg/dL)	90.0 ± 7.72	92.2 ± 7.60	0.0076
2hrPG (mg/dL)	124 ± 28.5	125 ± 23.4	0.62
Total fat mass (kg)	31.7 ± 13.9	29.1 ± 12.9	0.10
Trunk fat mass (kg)	15.4 ± 7.51	14.7 ± 6.95	0.43
Fasting insulin (μU/mL)	7.54 ± 5.89	8.21 ± 8.03	0.38
Total cholesterol (mg/dL)	174 ± 33.2	179 ± 32.0	0.22
HDL (mg/dL)	53.5 ± 14.9	52.1 ± 13.4	0.40
Triglycerides (mg/dL)	79 ± 37.3	113 ± 64.6	<0.0001

*To convert glucose to millimoles per liter multiply by 0.05551; to convert insulin to picomoles per liter multiply by 6; to convert total and HDL cholesterol to millimoles per liter multiply by 0.0259; and to convert triglycerides to millimoles per liter multiply by 0.0133*.

### Leptin Levels

**Figure 1:**
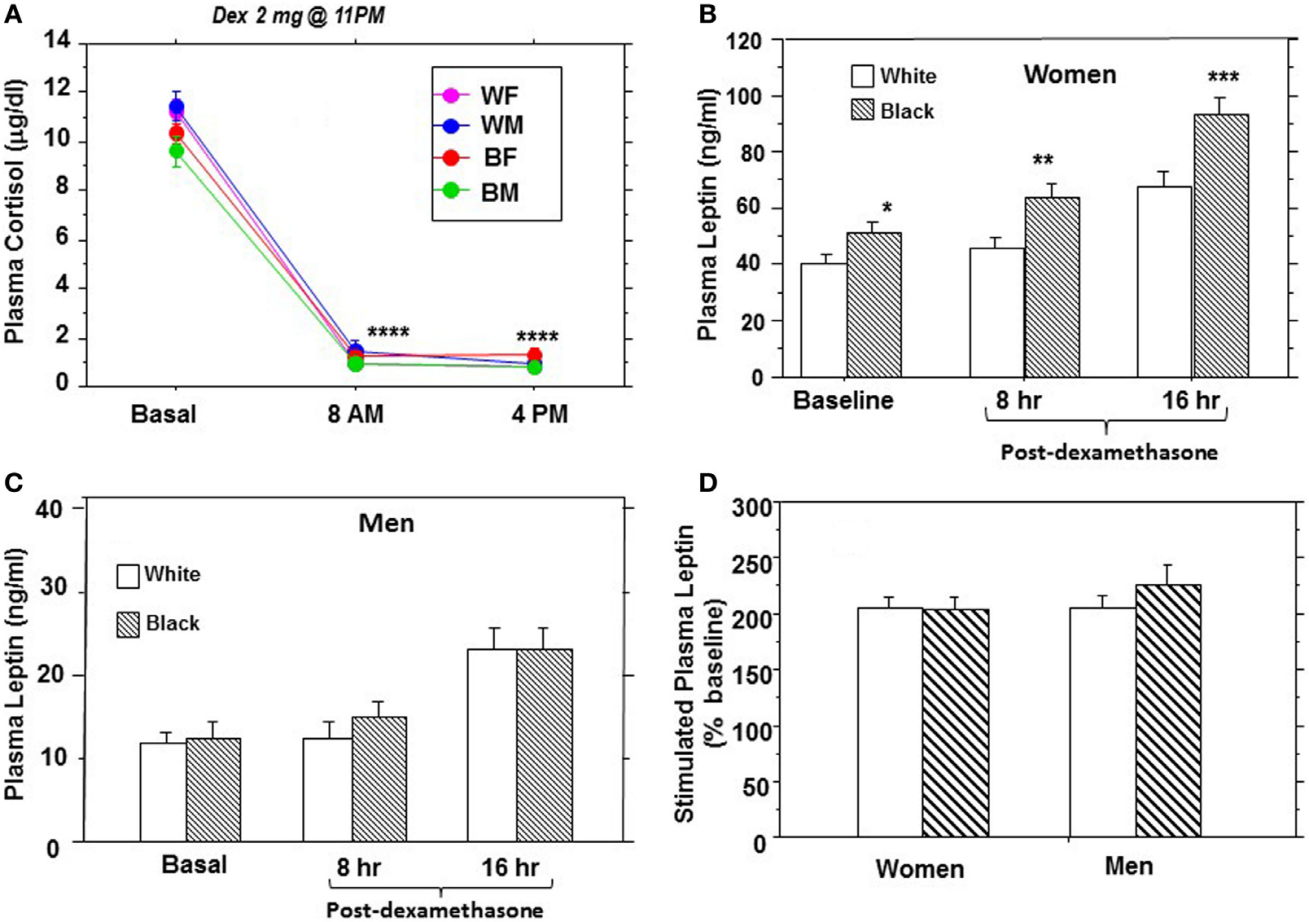
Plasma cortisol **(A)** and leptin **(B–D)** responses to a single dose (2 mg) of dexamethasone in African-American and European-American men and women. Abbreviations: WF, white female; WM, white male; BF, black female; BM, black male (*P = 0.004, **P = 0.0036, ***P = 0.0024, and ****P < 0.0001).

Owing to the known gender dimorphism in circulating leptin levels, plasma leptin data are presented separately for men and women (Table 2; Figure 1). The mean fasting plasma leptin level was higher in black women than white women (50.6 ± 47.7 vs. 39.5 ± 36.7 ng/mL, *P* = 0.004) but similar in black men and white men (12.4 ± 13.9 vs. 11.1 ± 9.57 ng/mL, *P* = 0.59) (Table 2). Following ingestion of the 2-mg dose of dex, the mean plasma cortisol levels were suppressed to <1.5 μg/dL in all subject groups at the 8- and 16-h sampling times (Figure 1A; Table 2). The sustained suppression of plasma cortisol levels reflects feedback inhibition of corticotropin release by exogenous glucocorticoid, which is *prima facie* evidence of dex ingestion by study participants.

**Table 2:** Plasma cortisol and leptin levels before and after dexamethasone (dex) (2 mg).^a^

	Black subjects	White subjects	*P*-value
Baseline cortisol (μg/dL)	10.1 ± 4.54	11.3 ± 4.69	0.03
8:00 a.m. day 2 cortisol (μg/dL)	1.18 ± 1.46	1.10 ± 1.52	0.66
4:00 p.m. day 2 cortisol (μg/dL)	1.17 ± 2.63	0.90 ± 0.71	0.72
**Fasting leptin (ng/mL)**			
Women	50.6 ± 47.7	39.5 ± 36.7	0.004
Men	12.4 ± 13.9	11.1 ± 9.57	0.59
**Peak leptin (ng/mL)**			
Women	93.6 ± 65.4	67.9 ± 54.1	0.0024
Men	23.0 ± 19.5	22.9 ± 17.9	0.99
**Delta leptin (%, ±SEM)**			
Women	103 ± 10.7	105 ± 8.51	0.99
Men	126 ± 20.3	105 ± 9.83	0.09

*^a^Baseline fasting blood sample was drawn at 8:00 a.m. on day 1 and dex (2 mg) was administered between 11:00 p.m. and midnight on day 1, followed by blood sampling at 8:00 a.m. and 4:00 p.m. on day 2. Peak leptin response occurred at 4:00 p.m*.

As previously reported by us (14, 16), the peak plasma leptin response to dex occurred at 16 h following ingestion, although increased secretion was evident at 8 h (Figures 1B,C). The baseline difference in fasting leptin levels between black women and white women was exaggerated following dex stimulation (Figure 1B), but the responses among men did not differ significantly by race (Figure 1C). To account for baseline differences in individual leptin levels, we expressed stimulated responses as a percentage of baseline values before comparing distinct groups. The peak leptin response to dex (% baseline, ±SEM) was 216 ± 9.04% in black participants vs. 204 ± 6.74% in white participants (*P* = 0.19), and 217 ± 9.1% in men vs. 204 ± 6.60% in women (*P* = 0.11). The peak plasma leptin responses to dex showed a consistent doubling from baseline (~200% baseline) in all four demographic groups: black men and white men; black women and white women (Figure 1D). The mean (±SEM) incremental change in leptin from baseline was 103 ± 10.7 vs. 105 ± 8.51% in black vs. white women (*P* = 0.99) and 126 ± 20.3 vs. 105 ± 9.83% in black men vs. white men (*P* = 0.09) (Table 2).

**Figure 2:**
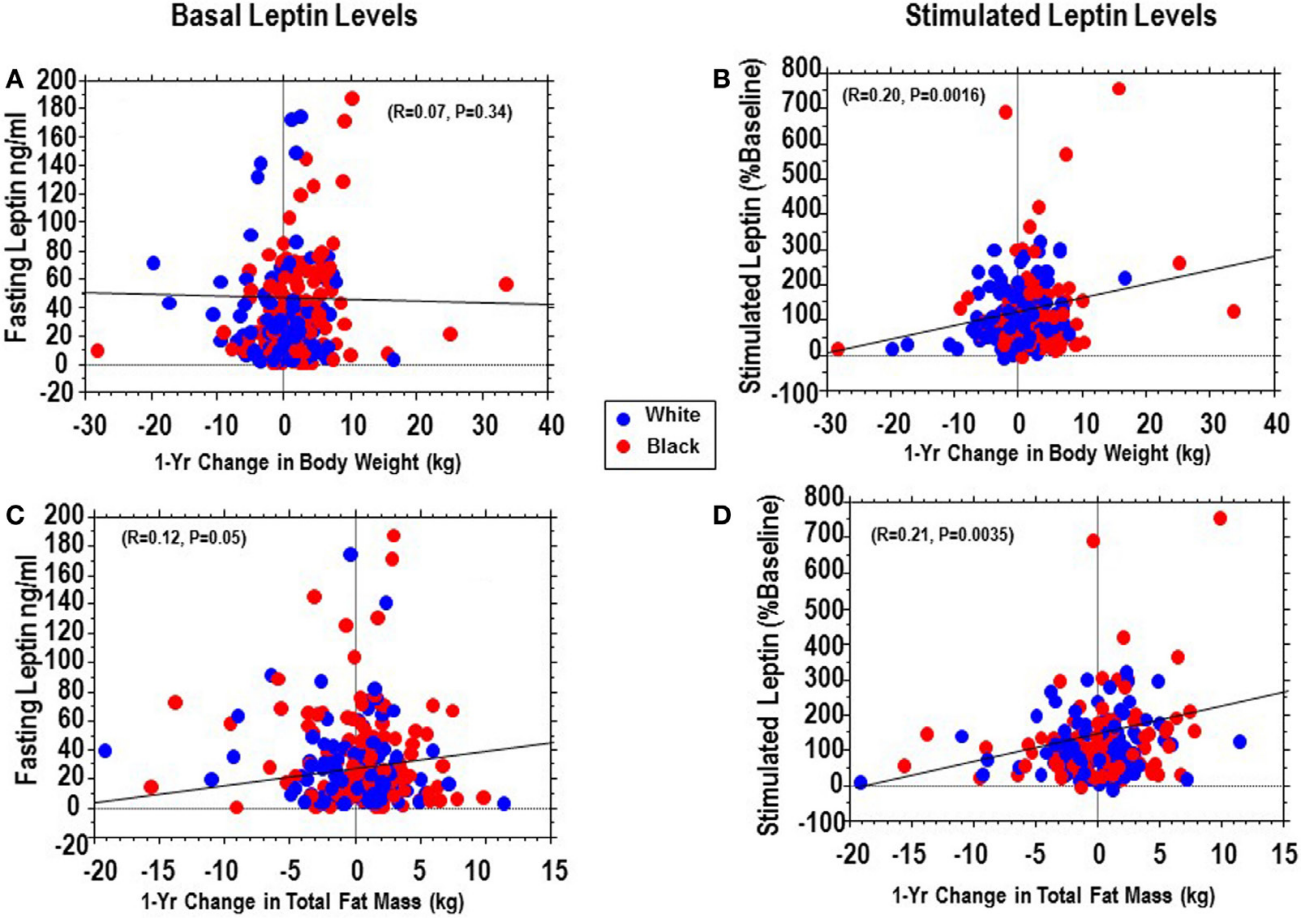
Regression of basal (fasting) plasma leptin vs. 1-year change in weight **(A)** or total fat **(C)** compared with regression of stimulated plasma leptin vs. and 1-year change in weight **(B)** or total fat **(D)** in African-Americans (red dots) and European-Americans (blue dots).

Being a natural history study, our POP-ABC participants did not receive any behavioral or pharmacological intervention for control of body weight or alteration of glucose levels. These free-living participants maintained their usual habits and often exhibited fluctuations in weight and body composition during serial measurement. We calculated the 1-year changes in weight and total body fat mass from enrollment (baseline) and evaluated the association of fasting and stimulated leptin secretion (assessed in year 1) with prospective changes in weight and fat mass in year 2. We found that fasting leptin levels had no significant relationship with 1-year change in weight (*P* = 0.34) and only a marginal relationship with 1-year changes total fat mass (*P* = 0.05) (Figures 2A,B). By contrast, peak stimulated leptin levels were modestly but significantly associated with 1-year trajectories in weight (*r* = 0.20, *P* = 0.0016) and total fat mass (*r* = 0.21, *P* = 0.0035) (Figures 2C,D).

#### Percentile Analysis and Cardiometabolic Correlates

**Figure 3:**
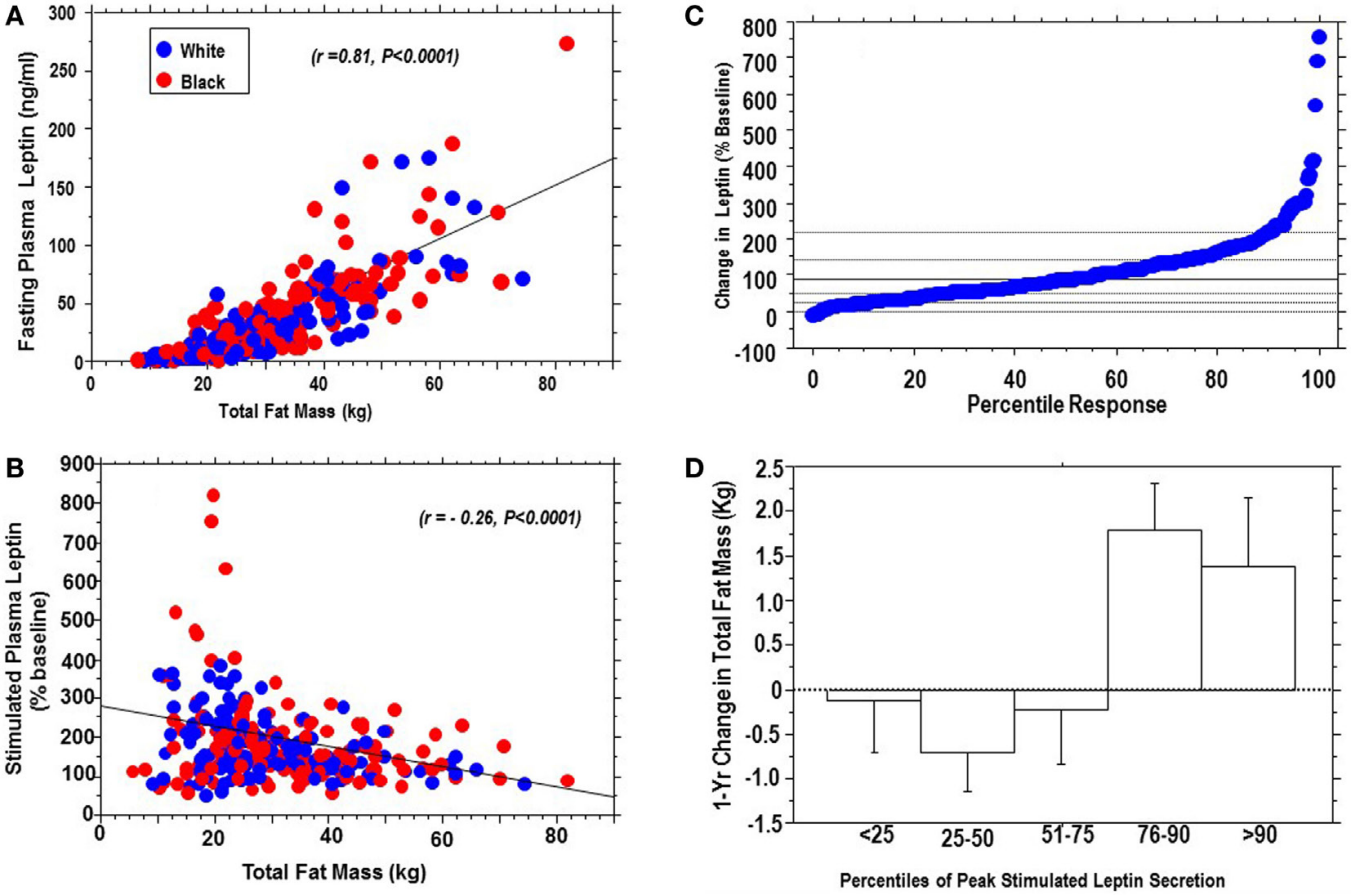
Positive association of fasting plasma leptin levels with body fat **(A)**, and inverse association of stimulated plasma leptin levels with body fat **(B)**, in African-Americans (red dots) and European-Americans (blue dots); percentile distribution of percent change in leptin following dexamethasone **(C)** and association with 1-year change in total fat mass **(D)**. There was a significant interaction between change in leptin and change in fat mass, *P* = 0.0006.

Fasting leptin levels correlated positively with body fat (*r* = 0.81, *P* < 0.0001), whereas peak stimulated leptin levels correlated inversely with body fat (*r* = −0.26, *P* < 0.0001) (Figures 3A,B). Figure 3C shows the percentile plot for change in leptin following dex ingestion for the entire cohort. The change in plasma leptin, expressed as a percentage of baseline levels, showed marked individual variability (range: −8 to +800%). Sensitivity analysis showed that plasma leptin responses to dex in 29 subjects (15 black, 14 white) were at or above the 90th percentile, and 74 subjects (38 black, 36 white) gave responses below the 25th percentile of the cohort distribution. There was a significant interaction between percentile response and 1-year change in body weight (*ANOVA P* = 0.0058) and total fat mass (*ANOVA P* = 0.0006). Subjects below the 75th percentile of leptin response maintained stable (or decreasing) body weight and total fat mass, whereas those above the 75th percentile had an increased risk of weight gain (Figure 3D). In univariate models significant associations were observed between dynamic leptin response to dex and fasting leptin levels (*r* = −0.29, *P* < 0.0001), BMI (*r* = 0.26, *P* < 0.0001), HOMA-B (*r* = −0.26, *P* = 0.0027), HOMA-IR (*r* = −0.24, *P* = 0.01), triglycerides (*r* = −0.17, *P* = 0.0078), FPG (*r* = −0.13, *P* = 0.027), hsCRP (*r* = −0.13, *P* = 0.027), and systolic blood pressure (BP) (*r* = −0.12, *P* = 0.037). Age, race, and gender were not significant predictors of stimulated leptin responses.

## Discussion

Despite more than 20 years of investigation, the exact role of leptin in human metabolic pathophysiology remains unclear. Leptin replacement is remarkably effective in treating rare forms of human obesity due to mutation of the leptin gene (4, 5). However, hopes of leptin therapy for common forms of obesity have not been fulfilled, although recombinant human leptin received approval by the U.S. Food and Drug Administration in 2014 for the treatment of generalized lipodystrophy (7, 24). The physiology and regulation of leptin also need to be fully clarified, as the notion of leptin as an anorectic, anti-obesity hormone has been expanded to emphasize leptin’s role as a rapid signal of starvation or energy deficit (25). Our previous studies focusing on the hormonal regulation of leptin in humans have established glucocorticoids as potent leptin secretagogues and have proposed that the glucocorticoid–leptin axis might be of metabolic relevance (14, 16, 18, 19, 26–28). The possible physiologic significance of glucocorticoid–leptin interaction is suggested by four related observations. First, the plasma leptin response to glucocorticoid has been demonstrated at physiological doses of hydrocortisone in humans (19). Second, glucocorticoid-induced hyperleptinemia is abolished by fasting (19, 29). Thirdly, inhibition of steroidogenesis by metyrapone decreases leptin production in humans (28, 30). Fourthly, glucocorticoid-induced hyperleptinemia has been reported to modulate anti-ingestive behavior in humans (27).

This report was an exhaustive analysis of basal and dynamic leptin secretion in a biracial cohort of otherwise healthy offspring of parents with T2D. Our findings confirm the well-known gender dimorphism in leptin levels (~3-fold higher in women than men). Although black women had higher basal and stimulated leptin levels than white women, the difference is accounted for by the higher BMI and fat mass among the former. Thus, the correlation between fasting leptin and body fat was identical (*r* = 0.81) in black and white subjects. Using a modification of our previous method, we demonstrated that the lower dose of dex (2 mg) stimulated leptin secretion by approximately the same magnitude compared with higher glucocorticoid doses (14, 16, 18). Our findings show definitively that a doubling of basal leptin levels is the expected peak response to glucocorticoid among healthy adults. Furthermore, there is no discernible evidence of gender or ethnic disparities when dynamic leptin secretion is expressed as a percentage of baseline values.

We observed an inverse correlation between stimulated leptin levels and total body fat, which contrasts with the well-recognized direct correlation between fasting leptin levels and adiposity. The mechanism for the inverse relationship is unclear. We propose that people with high body fat might be near maximal in their leptin secretory potential and, thus, limited in their capacity for mounting fold increases in leptin secretion (31). By contrast, people with lower body fat (and therefore low basal leptin levels) might be more capable responding more briskly to a response to leptin secretagogue. Another novel finding from the present study is the dissociation between basal and stimulated leptin levels in the prediction of future weight gain in our biracial cohort of African-Americans and European-Americans. A previous report had suggested that fasting plasma leptin levels might predict future weight gain in Pima Indians (32). That report was based on an analysis of fasting plasma leptin levels in 19 Pima Indians who gained weight and 17 who maintained their weight during approximately 3 years of follow-up (32). In our much larger sample of 254 subjects, we did not observe a significant association between fasting plasma leptin levels and future weigh change over a 1-year period. By contrast, we noted a weak but significant association between stimulated leptin levels and 1-year weight gain. We also found an inverse association between stimulated leptin levels and body fat at baseline. The latter finding suggests that the magnitude of leptin response to secretagogue might be a surrogate for adipocyte size and capacity for additional fat storage. Viewed in that light, obese, hyperleptinemic individuals who give a weak response to glucocorticoid stimulation might be signaling a plateau in their weight trajectory and adipocyte fat storage capacity (31). By contrast, heterogeneity in dynamic leptin responses among leaner individuals might convey information regarding differential adipocyte secretory/fat storage capacity, with implications for future weight gain. Indeed, our percentile and sensitivity analyses showed that individuals below the 75th percentile of stimulated leptin secretion maintained stable weight and body fat, whereas hypersecretors above the 75th percentile were more likely to experience weight gain. Further studies correlating dynamic leptin secretory status with morphological characteristics of adipocytes from fat biopsies might be informative. Similarly, longitudinal studies evaluating dynamic leptin secretion in relation to clinical cardiometabolic endpoints would also be valuable.

In conclusion, findings from our biracial cohort indicate that basal and dynamic (dex-stimulated) leptin levels were similar in African-Americans and Caucasians. Moreover, dynamic leptin levels correlated with a wide range of cardiometabolic risk markers (including blood glucose, insulin, BP, triglycerides, hsCRP and short-term weight trajectories).

## Ethics Statement

The POP-ABC study protocol was approved by the University of Tennessee Health Science Center Institution Review Board. All subjects gave written informed consent before the initiation of the study, which was conducted in accordance with the principles of the Declaration of Helsinki.

## Author Contributions

The authors’ individual contributions included the following: SD-J as the principal investigator, developed the study concept and design and wrote the manuscript; SE and CE collected the data and reviewed and revised the manuscript; and IO conducted the statistical analysis and reviewed and revised the manuscript.

## Conflict of Interest Statement

The authors declare that the research was conducted in the absence of any commercial or financial relationships that could be construed as a potential conflict of interest.

## Abbreviations

ANOVA, analysis of variance; BMI, body mass index; BP, blood pressure; Dex, dexamethasone; DEXA, dual energy X-ray absorptiometry; FPG, fasting plasma glucose; HOMA-B, homeostasis model of beta-cell function; HOMA-IR, homeostasis model of insulin resistance; hsCRP, high-sensitivity C-reactive protein; NGT, normal glucose tolerance; OGTT, oral glucose tolerance test; POP-ABC, Pathobiology of Prediabetes in a Biracial Cohort; 2hrPG, two-hour post-load plasma glucose during 75-gram standard oral glucose tolerance test; T2D, type 2 diabetes.
